# Reduced Susceptibility to Rifampicin and Resistance to Multiple Antimicrobial Agents among *Brucella abortus* Isolates from Cattle in Brazil

**DOI:** 10.1371/journal.pone.0132532

**Published:** 2015-07-16

**Authors:** Rebeca Barbosa Pauletti, Ana Paula Reinato Stynen, Juliana Pinto da Silva Mol, Elaine Maria Seles Dorneles, Telma Maria Alves, Monalisa de Sousa Moura Souto, Silvia Minharro, Marcos Bryan Heinemann, Andrey Pereira Lage

**Affiliations:** 1 Escola de Veterinária, Universidade Federal de Minas Gerais, Belo Horizonte, Minas Gerais, Brazil; 2 Escola de Medicina Veterinária e Zootecnia, Universidade Federal do Tocantins, Araguaína, Tocantins, Brazil; 3 Faculdade de Medicina Veterinária e Zootecnia, Universidade de São Paulo, São Paulo, Brazil; Universidad Nacional de La Plata., ARGENTINA

## Abstract

This study aimed to determine the susceptibility profile of Brazilian *Brucella abortus* isolates from cattle to eight antimicrobial agents that are recommended for the treatment of human brucellosis and to correlate the susceptibility patterns with origin, biotype and MLVA16-genotype of the strains. Screening of 147 *B*. *abortus* strains showed 100% sensitivity to doxycycline and ofloxacin, one (0.68%) strain resistant to ciprofloxacin, two strains (1.36%) resistant to streptomycin, two strains (1.36%) resistant to trimethoprim-sulfamethoxazole and five strains (3.40%) resistant to gentamicin. For rifampicin, three strains (2.04%) were resistant and 54 strains (36.73%) showed reduced sensitivity. Two strains were considered multidrug resistant. In conclusion, the majority of *B*. *abortus* strains isolated from cattle in Brazil were sensitive to the antimicrobials commonly used for the treatment of human brucellosis; however, a considerable proportion of strains showed reduced susceptibility to rifampicin and two strains were considered multidrug resistant. Moreover, there was no correlation among the drug susceptibility pattern, origin, biotype and MLVA16-genotypes of these strains.

## Introduction

Brucellosis is a worldwide-distributed zoonosis caused by bacteria of the genus *Brucella*. In cattle, it is mainly caused by *Brucella abortus* and it is responsible for a significant impact on the economy and public health, especially in developing countries [1]. Bovine brucellosis transmission to human occurs mainly by contact with infected animals or through the consumption of contaminated foods [1,2].

Veterinarians, breeders, slaughterhouse workers, and people in close contact with cattle are the group with higher risk of infection. They are direct exposed to material resulting from abortions and secretions from infected animals and carcasses, which are the most common infection routes in at-risk groups [1,3]. Furthermore, they can also be accidentally contaminated by the live *B*. *abortus* vaccine strains [4]. Human brucellosis presents nonspecific symptoms; therefore, it is important for physicians to be informed that the patient is in a risk groups and may have been exposed to the agent [2]. Moreover, the treatment requires a combined regimen of antibiotics and should be started as soon as possible after infection for maximal effectiveness [2]. Additionally, because *Brucella* spp. are intracellular pathogens, the treatment must be of long duration and with agents that efficiently penetrate macrophages [5].

The drugs commonly recommended for the treatment of human brucellosis, usually used in dual or triple regimens, are doxycycline, streptomycin, gentamycin, rifampicin, tetracycline and co-trimoxazole (trimethoprim plus sulfamethoxazole) [1,6,7]. The most widely used and recommended regimens are those combining doxycycline and an aminoglycoside or rifampicin, however monotherapy and other combinations have also demonstrated some efficacy [6,7]. Thus, the determination of the antibiotic susceptibility profile of *B*. *abortus* is clinically important, because it may aid treatment decisions regarding using a single antimicrobial or combinations of antimicrobials according to the origin, genotype and phenotype characteristics of the infecting strain.

In Brazil, data on human brucellosis indicate that the disease occurs in various regions of the country, particularly in certain occupational groups, which work in direct contact with animals [3,8]. However, the literature regarding the antimicrobial susceptibility of B. abortus isolates from Brazil is scarce. Therefore, the aims of this study were to determine the susceptibility profile of B. abortus strains isolated from naturally infected cattle in Brazil between 1977 and 2009, to identify the most commonly used antimicrobial agents for the treatment of human brucellosis, and to assess the relationship among the susceptibility profiles of the strains with their origin, biotype and genotype.

## Material and Methods

### Bacterial strains

**Fig 1:**
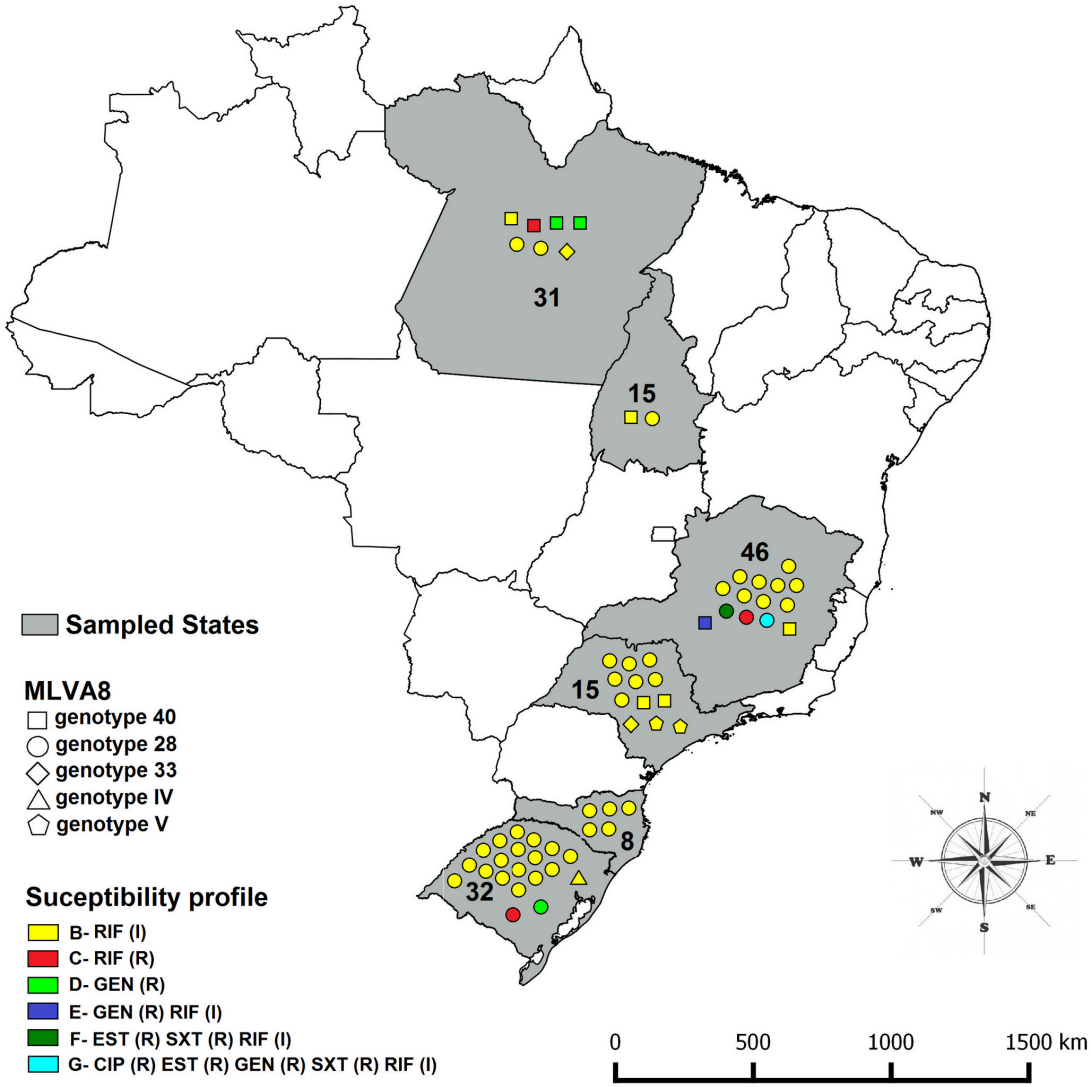
Resistance pattern, MLVA8 genotype and the spatial localization of the *B*. *abortus* strains that showed resistance or intermediate profile to at least one of the tested antibiotics. The states sampled are highlighted in gray. The number inside the state represents the number of sampled *B*. *abortus* strains from that state. The shape of the symbols used represents the MLVA8-genotype observed for the strains with resistant or intermediate profile. The color of the symbols represents the resistance pattern of the nine *B*. *abortus* that were resistant or intermediate to at least one of the tested antimicrobials, according to the Table 2. CIP: ciprofloxacin; DOX: doxycycline; EST: streptomycin; GEN, gentamicin; OFX, ofloxacin; RIF, rifampicin; SXT, trimethoprim-sulfamethoxazole. (R): resistant; (I): intermediate.

One hundred and forty-seven *B*. *abortus* strains isolated from naturally infected cattle in Brazil between 1977 and 2009 [9] were tested. The strains were previously isolated [9] from specimens collected from animal that were slaughter under humane conditions and under official inspection service of the Ministry of Agriculture, Livestock and Food Supply. All bacteriological analyses were performed following the Brazilian program on the control and eradication of brucellosis and tuberculosis. Three *B*. *abortus* reference strains, *B*. *abortus* ATCC 23448^T^ = 544, S19 vaccine, and 2308 strains were also included in the present study. The field strains were isolated from the States of Minas Gerais (46), Pará (31), Rio Grande do Sul (32), Santa Catarina (8), São Paulo (15), and Tocantins (15) [9] (Fig 1).

### Strain identification

Field isolates were submitted to classical identification tests: nitrate reduction, catalase, oxidase, citrate, urease, CO_2_ requirement, H_2_S production, inhibition of growth by basic fuchsin and thionin, and agglutination with monospecific antisera [10]. Based on their phenotypic characteristics, strains were classified into biovar. All strains were confirmed as belonging to the genus *Brucella* by genus-specific PCR [11], and as *B*. *abortus* by AMOS-PCR [12] and Bruce-ladder multiplex PCR [13].

### Antimicrobial susceptibility testing

Minimum Inhibitory Concentration (MIC) using agar dilution was determined according to the Clinical and Laboratory Standards Institute (CLSI) M45-A2 manual [14] for amikacin (Eurofarma, São Paulo, Brazil), ciprofloxacin (DEG, Hong Kong, China), doxycycline (Sigma-Aldrich, St. Louis, USA), streptomycin (Sigma-Aldrich, St. Louis, USA), gentamicin (Galena, Campinas, Brazil), ofloxacin (Sigma-Aldrich, St. Louis, USA), rifampicin (Lupin, Mumbai, India) and trimethoprim (Genix Pharma, Karachi, Pakistan) plus sulfamethoxazole (Fluka, Saint Louis, USA) (19 parts of sulfamethoxazole to 1 part trimethoprim) in 12 two-fold dilutions from 0.06 μg / mL to 128 μg / mL. Briefly, Mueller-Hinton agar (Difco, Detroit, USA) plates plus the antimicrobial concentrations tested were inoculated with bacterial suspensions adjusted to a turbidity equivalent of 0.5 McFarland standard and incubated for 48 h at 37°C and 5% CO_2_ [14].

Determination of the MICs was performed in duplicate and all antibiotics were tested with the reference strains: *Escherichia coli* ATCC 25922, *Enterococcus faecalis* ATCC 29212, *Pseudomonas aeruginosa* ATCC 27853, *Staphylococcus aureus* ATCC 29213 and *Streptococcus pneumoniae* ATCC 49619 to ensure that the results were within acceptable limits of quality control for susceptibility testing [15]. Furthermore, in all assays, *E*. *coli* ATCC 25922, *S*. *aureus* ATCC 29213 and *B*. *abortus* 544 = ATCC 23448^T^ were used as growth quality controls. As growth controls, two Mueller-Hinton agar plates, without antibiotics, were employed at the beginning of the antibiotic plating sequence and at the end of this sequence.

MIC_50_ and MIC_90_ levels were defined as the lowest concentration of the antibiotic at which 50% and 90% of the strains were inhibited, respectively. The resistance breakpoints were set in accordance with the CLSI M100-S17 document [15] for *Brucella* spp. or slow-growing bacteria (*Haemophilus* spp.). Based on these criteria the strains were classified as resistant or sensitive to antimicrobials. However, as amikacin did not have a defined breakpoint, it was determined by its MIC_50_, MIC_90_ and MIC range. For rifampicin the strains were also classified as intermediate susceptible.

### MLVA16 Genotyping

All of the studied *B*. *abortus* strains were genotyped by MLVA16 [9]. Briefly, DNA from each strain was submitted to the multiple *loci* VNTR analyses (MLVA-typing) according to Le Flèche et al. [16], using sixteen primers for different VNTR *loci* in *Brucella* spp. (MLVA16) [16]. Band size estimates were converted into number of repeat units for each *locus* [16], using the BioNumerics 5.1 software (Applied Maths, Sint-Martens-Latem, Belgium). Clustering analysis also was performed with the BioNumerics 5.1 software based on UPGMA [16]. One hundred thirty-seven field strains used in the present study were previously genotyped [9].Genotypes obtained were compared to those deposited in the MLVAbank 2012 for Bacterial Genotyping (http://minisatellites.u-psud.fr/MLVAnet/).

## Results

All isolates were identified as *B*. *abortus* by biochemical tests and PCR. The MIC_50_, MIC_90_, and MIC range values found for 147 *B*. *abortus* studied strains, as well as the defined breakpoints and the number of resistant strains to each antimicrobial agent are shown in Table 1. All duplicates showed equal MIC results. Ofloxacin and doxycycline were the antibiotics that showed better activities against the Brazilian *B*. *abortus* cattle isolates; 100% of the strains tested were sensitive to these antimicrobials (Table 1). Only one of the *B*. *abortus* strain (0.68%) showed resistance to ciprofloxacin, whereas three (2.04%) and five (3.40%) strains were resistant to rifampicin and gentamicin, respectively (Table 1). The three tested reference strains (544, S19 and 2308) were susceptible to the seven antimicrobials, which have established breakpoints. The MIC results for all the antimicrobial agents against *B*. *abortus* reference strains are shown in the S1 Table.

**Table 1:** Minimal Inhibitory Concentration (MIC_50_, MIC_90_ and range) values of *Brucella abortus* strains isolated from cattle in Brazil, 1977–2009, to eight antimicrobials used in the treatment of human brucellosis.

Antimicrobial agent	MIC range^a^	MIC_50_ ^b^		MIC_90_ ^c^		Resistance		
		Value	no.^d^	Value	no.	CP^e^	no.^f^	%^g^
Amikacin	2.0–≥256 (8)	4.0	97	8.0	144	–	–	–
Ciprofloxacin	0.5–2.0 (3)	0.5	146	0.5	146	>1.0	1	0.68
Doxycycline	≤0.06–2,0 (6)	0.5	133	0.5	133	>1.0	0	0
Streptomycin	0.125–≥256 (12)	2.0	141	2.0	141	>16	2	1.36
Gentamicin	0.25–≥256 (11)	1.0	141	1.0	141	>4.0	5	3.40
Ofloxacin	0.5–1.0 (2)	0.5	142	0.5	142	>2.0	0	0
Rifampin	0.125–8.0 (7)	1.0	90	2.0	144	≥4.0^h^	3	2.04
Trimethoprim-sulfamethoxazole	0.1/1.9–12.8/243.2 (8)	0.8/15.2	134	0.8/15.2	134	>2/38	2	1.36

^a^Variation of MIC values (number of dilution variations) for every antimicrobial

^b^Minimum Inhibitory Concentration required to inhibit the growth of 50% of the strains

^c^Minimum Inhibitory Concentration required to inhibit the growth of 90% of the strains

^d^Number of sensitive strains in the MIC established

^e^Breakpoint of resistance for *Brucella* spp. strains (μg/mL)

^f^Number of resistant strains

^g^Percentage of resistant strains

^h^The breakpoints for rifampin are: resistance ≥ 4.0 μg/mL, intermediate 2.0 μg/mL and sensitivity ≤ 1.0 μg/mL.

The susceptibility profiles for the strains tested against the seven antimicrobials are shown in the Table 2. This classification was created for grouping strains with similar susceptibilities to antimicrobials and then for facilitating the identification of the number of strains with resistant, intermediate and sensitive profiles. According to Magiorakos et al. [17], strains that were considered non-susceptible to at least one agent in three different antimicrobial classes were classified as multidrug resistant. Two *B*. *abortus* strains (1.36%) showed multidrug resistance, with the profile F resistant to one aminoglycosides (streptomycin), one sulfonamide (trimethoprim-sulfamethoxazole) and intermediate resistance to rifampicin, and profile G resistant to one quinolone (ciprofloxacin), two aminoglycosides (streptomycin and gentamicin), one sulfonamide (trimethoprim-sulfamethoxazole) and intermediate resistance to rifampicin (Table 2).

**Table 2:** Susceptibility profile of *Brucella abortus* strains isolated from cattle in Brazil, 1977–2009.

Antibiotics^a^							Profile^b^	no.^c^
CIP	DOX	EST	GEN	OFX	RIF	SXT		
S^d^	S	S	S	S	S	S	A	87
S	S	S	S	S	**I** ^e^	S	B	51
S	S	S	S	S	**R** ^f^	S	C	03
S	S	S	**R**	S	S	S	D	03
S	S	S	**R**	S	**I**	S	E	01
S	S	**R**	S	S	**I**	**R**	F	01
**R**	S	**R**	**R**	S	**I**	**R**	G	01

^a^ CIP: ciprofloxacin, DOX: doxycyclin, EST: streptomycin, GEN, gentamicin, OFX, ofloxacin, RIF: rifampicin, SXT, trimethoprim-sulfamethoxazole

^b^Susceptibility profiles to seven antimicrobials tested

^c^Number of strains with identical susceptibility profile

^d^Susceptible

^e^Intermediate

^f^Resistant.

Antibiotic susceptibility pattern, MLVA8 genotype and the spatial localization of the *B*. *abortus* strains that showed resistance or intermediate profile to at least one of the tested antibiotics are shown in the Fig 1. Analysis of the susceptibility profile and origin of the strains tested showed that four strains from Minas Gerais State (MG) [8.69% (4 / 46)] presented some pattern of resistance and that twelve [26.08% (12 / 46)] showed intermediate resistance to rifampicin. Among the four resistant strains (30, 61, 235 and 393.11), two were multi-resistant (30 and 393.11). Of the 31 strains from Pará (PA), three (strains A6, 164 and 198) [9.67% (3 / 31)] were resistant to just one antibiotic (gentamycin or rifampin) and four [12.90% (4 / 31)] presented an intermediate resistance profile to rifampicin. Of the 32 isolates from Rio Grande do Sul State (RS), two [6.25% (2 / 32)] were resistant to at least one antimicrobial and nineteen [59.37% (19 / 32)] showed an intermediate resistance to rifampicin. Among the isolates from the States of Santa Catarina (SC), São Paulo (SP) and Tocantins (TO) no resistant strains were found, but five [62.50% (5 / 8)] strains isolated from Santa Catarina, twelve [80.00% (12 / 15)] from São Paulo and two [13.33% (2 / 15)] from Tocantins showed intermediate resistance to rifampicin.

Distribution of the resistant, intermediate and susceptible profiles for the 147 *B*. *abortus* strains according to the year of isolation for all antimicrobial agents tested are shown in the Table 3. Information of origin, antimicrobial susceptibility pattern, biotype and MLVA16 genotype for all isolates are summarized in S2 Table. No association between those variables and the susceptibility profile of the tested *B*. *abortus* strains was observed.

**Table 3:** Distribution of the susceptibility profile to seven antimicrobials for the 147 *B*. *abortus* strains isolated from cattle in Brazil, 1977–2009, according to the year of isolation.

Isolation Year / Antimicrobial	Ciprofloxacin		Doxicicline		Streptomycin		Gentamicin		Ofloxacin		Rifampin			Trimethoprim-sulfamethoxazole		Total
	S^a^	R^b^	S	R	S	R	S	R	S	R	S	I^c^	R	S	R	
1977	3	-	3	-	3	-	3	-	3	-	1	2	-	3	-	3
1996	1	-	1	-	1	-	1	-	1	-	-	1	-	1	-	1
2002	5	-	5	-	5	-	5	-	5	-	3	2	-	5	-	5
2003	3	-	3	-	3	-	3	-	3	-	1	2	-	3	-	3
2004	8	-	8	-	8	-	7	1	8	-	5	3	-	8	-	8
2005	1	-	1	-	1	-	-	1	1	-	-	1	-	1	-	1
2006	26	1	27	-	26	1	26	1	27	-	18	8	1	26	1	27
2007	13	-	13	-	13	-	12	1	13	-	7	6	-	13	-	13
2008	48	-	48	-	48	-	47	1	48	-	38	9	1	48	-	48
NK^d^	38	-	38	-	37	1	38	-	38	-	17	20	1	37	1	38
Total	146	1	147	0	145	2	142	5	147	0	90	54	3	145	2	147

^a^ Suceptible

^b^ Resistant

^c^ Intermediate

^d^ Not known

## Discussion

To our knowledge, this is the first report describing the identification of multidrug resistant strains of *B*. *abortus*. Furthermore, a considerable proportion of the strains (36.73%) showed reduced susceptibility to rifampin, a drug present in the majority of combinations chosen for the treatment of human brucellosis [1,6,7]. On the other hand, our results also showed that most of the *B*. *abortus* strains tested were sensitive to the eight antibiotics evaluated.

In Brazil, despite the absence of broader studies showing the most common species of *Brucella* involved in human infections, it is suggests that human cases of brucellosis are due to *B*. *abortus*, as *B*. *melitensis* was never isolated in the country and *B*. *abortus* is the most prevalent *Brucella* spp. in animals, especially cattle [18]. In the present study, screening of the 147 *B*. *abortus* strains isolated from naturally infected cattle in Brazil exhibited MIC_90_ similar to those already described for *Brucella* spp. isolated from Egypt, Mexico, Spain and Turkey [5,19–21] against ciprofloxacin, doxycycline and ofloxacin. However, MIC values for the aminoglycosides (amikacin, gentamicin and streptomycin), trimethoprim-sulfamethoxazole and rifampin exhibited a wide range, showing strains with MIC values much higher than the average for those antibiotics described in the literature. In addition, MIC values for these drugs were different from those previously described for *Brucella* spp. from Cyprus, Egypt, Greece, Mexico, Syria and Turkey [20–23], which may suggest the beginning of an adaptation or resistance of these isolates to these antimicrobials.

Interestingly, most of the resistant strains found in this study were resistant to antimicrobials that presented wider MIC ranges, with the exception of doxycycline. A wide range of variation in MIC values may favor the observation of resistant strains, since it demonstrates the heterogeneity of the study population. On the other hand, gentamicin, streptomycin and trimethoprim-sulfamethoxazole showed a large range in MIC values and also exhibited equal MIC_50_ and MIC_90_ values, as was also true for ciprofloxacin, doxycycline and ofloxacin. Similarly, rifampicin and amikacin exhibited a range of only one dilution between the MIC_50_ and MIC_90_. Taken together these results suggest the existence of a homogeneous population in terms of susceptibility profile to these antibiotics, since for growth inhibition of 50 or 90% of strains the same concentration of these antibiotics is required.

The high MIC_90_ values (up to 8 μg / mL) observed for amikacin indicates a tendency toward resistance among the Brazilian *B*. *abortus* isolates to this drug, which is also supported by the high MIC_50_ and wide range of MIC values, although the assessment of *B*. *abortus* susceptibility to amikacin cannot be performed because there is no established breakpoint for *Brucella* spp. or other fastidious bacteria to this drug Likewise, some *B*. *abortus* strains [7.48% (11 / 147)] showed a tendency for resistance to trimethoprim-sulfamethoxazole, because their MIC values were close to the breakpoint established for this antimicrobial. Lower MIC values for trimethoprim-sulfamethoxazole against *Brucella* spp. were observed in Egypt, Italy, Peru and Turkey [21,23–25], which contrasted with our results, but *Brucella* spp. from Mexico and Saudi Arabia showed higher rates of trimethoprim-sulfamethoxazole resistance [20,26]. These differences may be a reflection of the differences in the strains regarding to origin, host of isolation, time frame of the experiment, and / or due to differences among *Brucella* spp. species tested, since all those studies tested *B*. *melitensis* from humans.

Another important finding from this study was the apparently trend towards resistance to rifampicin observed in 36.73% (54 / 147) of the *B*. *abortus* strains from Brazil. This high frequency of reduced susceptibility to rifampicin among *B*. *abortus* isolates from Brazil can suggest the emergence of resistant strains to this drug, although the mechanism involved in this intermediate susceptibility needs further investigation. Association among the intermediate profile of rifampicin susceptibility and the year of isolation of the strains, confirming the temporal emergence of rifampicin resistance, is difficult to be made, as the number of isolates increased during the studied period (Table 3). Therefore, it is tempting to speculate that the intermediate profile to rifampicin observed in the present studied is not new and could explain some failures and relapse in the treatment that may have occurred. However, although the data do not support the idea of a temporal emergence, it is important to take into account that the three strains resistant to rifampicin were among the more recent isolates (2006 and 2008). Recently, in Egypt, it was also observed that there is a high rate of *B*. *melitensis* isolates [45.0% (158 / 355)] with reduced susceptibility to rifampicin [21]. One of the major concerns surrounding the emergence of resistant *Brucella* spp. is that rifampicin is one of the antibiotics of choice for the treatment of brucellosis in humans [1,6,7]. Furthermore, the Ministério da Agricultura, Pecuária, e Abastecimento (Ministry of Agriculture, Livestock and Food Supply) approved the use of RB51 for vaccination of adult cattle in the country since 2003. Thus, although rifampicin is widely used as an antibiotic for the treatment of human brucellosis, its use in Brazil should be recommended with caution in light of the high rate of suggested resistant *B*. *abortus* strains and the intrinsic resistance of RB51 to this antibiotic [1]. In addition, future susceptibility testing in *B*. *abortus* isolates is critical to monitor the emergence of rifampicin-resistant phenotypes and to guide the use of this antimicrobial in the treatment of human brucellosis in Brazil.

The preferred treatment in uncomplicated human brucellosis is doxycycline-aminoglycoside or a doxycycline-rifampicin combination [1,6,7]. However, an alternative treatment for human brucellosis uses a combination of doxycycline and ofloxacin, with or without the addition of another base [6]. The addition of a third base in this combination may be a safer alternative, given that it has been reported that the use of a quinolone with doxycycline or rifampicin is less effective than the combination of doxycycline with rifampicin or streptomycin [7]. In the present study, ofloxacin, ciprofloxacin and doxycycline were the most active drug against *B*. *abortus* (MIC_50/90_ values of 0.5 / 0.5 μg / mL); therefore, the treatment options suggested by Ariza et al. [6] or Skalsky et al. [7] seem to be the most suitable for the treatment of human brucellosis in Brazil. Moreover, 100% of the strains tested were susceptible to doxycycline and ofloxacin. Similar results have been reported for quinolones and tetracycline for *Brucella* spp. isolates from China, Egypt and Peru [21,25,27].

Additionally, the susceptibility profile observed in this study for isolates of *B*. *abortus* from cattle in Brazil has major clinical relevance for human brucellosis, since nine isolates (6.12%) showed resistance to one or more of the most important antibiotics recommended for the treatment of the disease in humans. Two of these strains were classified as multiresistant by showing resistance to two or more classes of antimicrobials (aminoglycosides, quinolones, sulfonamides and rifampicin), which could be the result of different mechanisms, such as efflux pump, which is a common mechanism of resistance to several antibiotics [28]. Antimicrobial transporters conferring resistance to several classes of antimicrobials such as tetracycline, macrolides, fluoroquinolones and aminoglycosides have been described in the literature [28], as have the existence of proteins capable of mediating drug resistance in *Brucella* spp. by an energy-dependent efflux mechanism [29]. Together these reports bolster the hypothesis that the development of multidrug resistance in strains of *B*. *abortus* is facilitated by an efflux pump mechanism.

In addition, although the present study did not investigate the mechanism of resistance associated with intermediate susceptibility and resistance to rifampicin profiles, it is possible that these phenotypes are associated with a mutation in the *rpoB* gene. Since Marianelli et al. [30] described a rifampicin resistance mechanism in *Brucella* spp. strains associated with mutations in the *rpoB* gene, which codifies the β subunit of DNA-dependent RNA polymerase, contain the site of action of rifampicin.

Our results also showed nine strains that were resistant to three of the four antimicrobials used as first-line treatment of human brucellosis (doxycycline, rifampin, streptomycin and gentamicin) [1,6,7]. These results have a special clinical interest, since therapy for the treatment of human brucellosis is a long process, requires a combination of drugs and presents high levels of relapses. Moreover, the treatment is usually based on experience gained over the years, instead of using an optimal therapy designed to each specific epidemiological situation. The present results, identifying resistance to aminoglycosides as well as multidrug resistance among *B*. *abortus* strains, can be explained in part by the pervasive use of these antimicrobials in many types of infections in cattle. The Brazilian veterinary antibiotic market generated sales of around USD 348 million in 2012 [31]and gentamicin (five resistant strains) is widely used in the treatment of mastitis and its association with clindamycin used as the gold standard in the treatment of postpartum endometritis [32]. Likewise, streptomycin (two resistant strains) is employed for the treatment of infections such as leptospirosis, diarrhea, mastitis and pneumonia [32], and is also widely used in livestock production. The easy access to these drugs and especially their numerous indications favor the indiscriminate use of these antimicrobials in cattle, which might have led to the development of resistance in the studied *B*. *abortus* strains.

In the present study, no association was found between biotypes or genotypes and the resistance profiles of *B*. *abortus* isolates from cattle in Brazil. Four different biovars were observed among the resistant strains, which represented a large degree of variation (S2 Table), since until now only five biovars (1, 2, 3, 4 and 6) were reported in Brazil [9,18]. In addition, the biovars of the resistant strains are the ones most frequently observed in the states where they were isolated [9] (biovar 1 from Minas Gerais and biovar 3 from Pará). Similar results were also observed for the panel 1 of MLVA16-genotyping (Fig 1 and S2 Table). Multidrug resistant *B*. *abortus* strains (profiles F and G) demonstrated identical genotypes in panel 1 and 2A, however those are the most frequent genotypes observed by those panels among isolates of *B*. *abortus* from Brazil [9]. The lack of association between genotypes and susceptibility profile of the strains became even clearer when analyses depicted in panels 2A and 2B were performed (S2 Table). Those panels showed a range of genotypes due to their higher variability [9,16]. Another factor that may have contributed to the absence of clustering of resistant strains by biotype or genotype was the low number of resistant strains (6.12%) among the *B*. *abortus* strains isolated from Brazil.

In conclusion, our findings show that the vast majority of *B*. *abortus* strains isolated from cattle in Brazil (138 / 147) were sensitive to antimicrobials commonly used for the treatment of human brucellosis. However, a considerable proportion (36.73%) showed reduced susceptibility to rifampin and two strains were considered multidrug resistant. No association among biotypes or genotypes and the resistance profiles of *B*. *abortus* isolates from Brazil was observed.

## Supporting Information

S1 Table Minimal Inhibitory Concentration (MIC) values of *Brucella abortus* reference strains (DOCX)

S2 Table Susceptibility pattern, genotype, biovar and epidemiological information of 147 *Brucella abortus* isolated from cattle in Brazil (DOCX)
