# Supplementary material for: Reduced Susceptibility to Rifampicin and Resistance to Multiple Antimicrobial Agents among Brucella abortus Isolates from Cattle in Brazil
Source: PLoS One. 2015 Jul 16;10(7):e0132532. doi: 10.1371/journal.pone.0132532 (PMC4504493; doi:10.1371/journal.pone.0132532)
Supplement: S1 Table — (DOCX) [file pone.0132532.s001.docx]

S1 Table - Minimal Inhibitory Concentration (MIC) values of *Brucella abortus* reference strains to seven antimicrobial agents used in the treatment of human brucellosis

| Strain | Minimal Inhibitory Concentration (µg/mL)^a^ | | | | | | | |
| --- | --- | --- | --- | --- | --- | --- | --- | --- |
|  | AMK | CIP | DOX | EST | GEN | OFX | RIF | SXT |
| S19 | 4 | 0.5 | 0.25 | 2 | 1 | 0.5 | 1 | 15.2-0.8 |
| 544 | 4 | 0.5 | 0.25 | 2 | 1 | 0.5 | 1 | 15.2-0.8 |
| 2308 | 4 | 0.5 | 0.5 | 1 | 1 | 0.5 | 0.5 | 7.6-0.4 |

^a^ AMK: amikacin; CIP: ciprofloxacin; DOX: doxycycline; EST: streptomycin; GEN, gentamicin; OFX, ofloxacin; RIF: rifampicin; SXT, trimethoprim-sulfamethoxazole
