# Supplementary material for: Reduced Susceptibility to Rifampicin and Resistance to Multiple Antimicrobial Agents among Brucella abortus Isolates from Cattle in Brazil
Source: PLoS One. 2015 Jul 16;10(7):e0132532. doi: 10.1371/journal.pone.0132532 (PMC4504493; doi:10.1371/journal.pone.0132532)
Supplement: S2 Table — (DOCX) [file pone.0132532.s002.docx]

S2 Table – Susceptibility pattern, genotype, biovar and epidemiological information of 147 *Brucella abortus* isolated from cattle in Brazil

| **Strain** | **Species** | **Biovar** | **Country** | **State** | **Isolation Year** | **CIP^a^** | **DOX^b^** | **EST^c^** | **GEN^d^** | **OFX^e^** | **RIF^f^** | **SXT^g^** | **P1^h^** | **P2A^i^** | **P2B^j^** | **MLVA 16** |
| --- | --- | --- | --- | --- | --- | --- | --- | --- | --- | --- | --- | --- | --- | --- | --- | --- |
| A1 | *B. abortus* | bv3 | Brazil | TO | 2004 | S | S | S | S | S | S | S | 40 | 2A1 | 2B1 | A |
| A4 | *B. abortus* | bv3 | Brazil | PA | 2004 | S | S | S | S | S | S | S | 40 | 2A1 | 2B2 | B |
| A6 | *B. abortus* | bv3 | Brazil | PA | 2004 | S | S | S | R | S | S | S | 40 | 30 | 2B3 | C |
| 30 | *B. abortus* | bv1 | Brazil | MG | 2006 | R | S | R | R | S | I | R | 28 | 33 | 2B4 | D |
| 31 | *B. abortus* | bv6 | Brazil | TO | NK | S | S | S | S | S | S | S | 40 | 2A9 | 2B22 | DI |
| 32 | *B. abortus* | bv1 | Brazil | MG | 2006 | S | S | S | S | S | S | S | 28 | 32 | 2B5 | AF |
| 33 | *B. abortus* | bv1 | Brazil | MG | 2006 | S | S | S | S | S | S | S | 28 | 33 | 2B4 | D |
| 34 | *B. abortus* | bv1 | Brazil | MG | 2006 | S | S | S | S | S | S | S | 28 | 33 | 2B4 | D |
| 35 | *B. abortus* | bv2 | Brazil | MG | 2006 | S | S | S | S | S | S | S | 28 | 2A2 | 58 | E |
| 36 | *B. abortus* | bv2 | Brazil | MG | 2006 | S | S | S | S | S | S | S | 28 | 2A2 | 58 | E |
| 37 | *B. abortus* | bv6 | Brazil | MG | 2006 | S | S | S | S | S | S | S | 40 | 32 | 2B1 | AG |
| 38 | *B. abortus* | bv2 | Brazil | MG | 2006 | S | S | S | S | S | S | S | 28 | 33 | 58 | AH |
| 39 | *B. abortus* | bv1 | Brazil | MG | 2006 | S | S | S | S | S | S | S | 28 | 33 | 2B4 | D |
| 40 | *B. abortus* | bv1 | Brazil | MG | 2006 | S | S | S | S | S | S | S | 28 | 33 | 2B4 | D |
| 42 | *B. abortus* | bv1 | Brazil | MG | 2006 | S | S | S | S | S | S | S | 28 | 33 | 2B5 | G |
| 43 | *B. abortus* | bv1 | Brazil | MG | 2006 | S | S | S | S | S | S | S | 28 | 33 | 2B5 | G |
| 44 | *B. abortus* | bv1 | Brazil | MG | 2006 | S | S | S | S | S | S | S | 28 | 33 | 2B5 | G |
| 45 | *B. abortus* | bv1 | Brazil | MG | 2006 | S | S | S | S | S | S | S | 28 | 30 | 2B6 | AI |
| 46 | *B. abortus* | bv1 | Brazil | MG | 2006 | S | S | S | S | S | S | S | 28 | 33 | 2B5 | G |
| 47 | *B. abortus* | bv2 | Brazil | MG | 2006 | S | S | S | S | S | S | S | 28 | 33 | 2B5 | G |
| 48 | *B. abortus* | bv1 | Brazil | MG | NK | S | S | S | S | S | S | S | 40 | 2A9 | 2B8 | DJ |
| 49 | *B. abortus* | bv2 | Brazil | MG | 2006 | S | S | S | S | S | S | S | 28 | 2A3 | 90 | AJ |
| 50 | *B. abortus* | bv2 | Brazil | MG | 2006 | S | S | S | S | S | S | S | 28 | 2A3 | 2B7 | AL |
| 54 | *B. abortus* | bv2 | Brazil | RS | 2006 | S | S | S | S | S | R | S | 28 | 2A3 | 91 | AM |
| 55 | *B. abortus* | bv2 | Brazil | RS | 2006 | S | S | S | S | S | S | S | 28 | 2A3 | 18 | AM |
| 61 | *B. abortus* | bv6 | Brazil | MG | 2005 | S | S | S | R | S | I | S | 40 | 2A4 | 2B8 | AO |
| 62 | *B. abortus* | bv3 | Brazil | MG | 2008 | S | S | S | S | S | S | S | 40 | 32 | 2B8 | H |
| 66 | *B. abortus* | bv3 | Brazil | MG | 2008 | S | S | S | S | S | S | S | 40 | 32 | 2B9 | AP |
| 70 | *B. abortus* | bv6 | Brazil | MG | 2007 | S | S | S | S | S | I | S | 40 | 32 | 2B10 | AQ |
| 95 | *B. abortus* | bv6 | Brazil | TO | 2007 | S | S | S | S | S | S | S | I | 2A5 | 2B11 | AR |
| 96 | *B. abortus* | bv1 | Brazil | TO | 2007 | S | S | S | S | S | I | S | 28 | 33 | 2B12 | AS |
| 136 | *B. abortus* | bv1 | Brazil | MG | 2007 | S | S | S | S | S | S | S | 28 | 33 | 2B13 | AT |
| 137 | *B. abortus* | bv1 | Brazil | MG | 2007 | S | S | S | S | S | I | S | 28 | 33 | 87 | AU |
| 138 | *B. abortus* | bv1 | Brazil | MG | 2007 | S | S | S | S | S | S | S | 28 | 33 | 2B14 | AV |
| 144 | *B. abortus* | bv1 | Brazil | MG | 2007 | S | S | S | S | S | S | S | 28 | 33 | 2B15 | AK |
| 147 | *B. abortus* | bv1 | Brazil | MG | 2007 | S | S | S | S | S | I | S | 28 | 33 | 87 | AX |
| 161 | *B. abortus* | bv6 | Brazil | PA | 2008 | S | S | S | S | S | S | S | 40 | 30 | 2B16 | AY |
| 162 | *B. abortus* | bv3 | Brazil | PA | 2008 | S | S | S | S | S | S | S | 40 | 30 | 2B17 | AW |
| 163 | *B. abortus* | bv3 | Brazil | TO | 2008 | S | S | S | S | S | S | S | II | 30 | 2B8 | AZ |
| 164 | *B. abortus* | bv3 | Brazil | PA | 2008 | S | S | S | R | S | S | S | 40 | 30 | 2B18 | BB |
| 165 | *B. abortus* | bv6 | Brazil | PA | 2008 | S | S | S | S | S | S | S | 40 | 30 | 2B19 | X |
| 166 | *B. abortus* | bv6 | Brazil | PA | 2008 | S | S | S | S | S | S | S | 40 | 32 | 2B20 | Y |
| 167 | *B. abortus* | bv6 | Brazil | PA | 2008 | S | S | S | S | S | S | S | 40 | 32 | 2B21 | BC |
| 168 | *B. abortus* | bv6 | Brazil | PA | 2008 | S | S | S | S | S | S | S | 40 | 32 | 2B20 | Y |
| 169 | *B. abortus* | bv6 | Brazil | TO | 2008 | S | S | S | S | S | S | S | 40 | 32 | 2B8 | H |
| 170 | *B. abortus* | bv6 | Brazil | PA | 2008 | S | S | S | S | S | S | S | 40 | 32 | 2B8 | H |
| 172 | *B. abortus* | bv6 | Brazil | PA | 2008 | S | S | S | S | S | I | S | 40 | 32 | 2B22 | U |
| 173 | *B. abortus* | bv6 | Brazil | PA | 2008 | S | S | S | S | S | S | S | 40 | 32 | 2B22 | U |
| 174 | *B. abortus* | bv3 | Brazil | PA | 2008 | S | S | S | S | S | S | S | 40 | 32 | 2B20 | Y |
| 175 | *B. abortus* | bv1 | Brazil | PA | 2008 | S | S | S | S | S | I | S | 33 | 33 | 2B23 | BD |
| 181 | *B. abortus* | bv2 | Brazil | TO | 2008 | S | S | S | S | S | S | S | 28 | 34 | 91 | BE |
| 182 | *B. abortus* | bv3 | Brazil | PA | 2008 | S | S | S | S | S | S | S | 40 | 8 | 2B19 | AB |
| 187 | *B. abortus* | bv3 | Brazil | TO | 2008 | S | S | S | S | S | S | S | 40 | 8 | 2B19 | AB |
| 188 | *B. abortus* | bv3 | Brazil | TO | 2008 | S | S | S | S | S | S | S | 40 | 8 | 2B19 | AB |
| 189 | *B. abortus* | bv3 | Brazil | PA | 2008 | S | S | S | S | S | S | S | 40 | 8 | 2B16 | BF |
| 190 | *B. abortus* | bv6 | Brazil | PA | 2008 | S | S | S | S | S | S | S | 40 | 8 | 2B19 | AB |
| 192 | *B. abortus* | bv3 | Brazil | PA | 2008 | S | S | S | S | S | S | S | 40 | 8 | 2B19 | AB |
| 194 | *B. abortus* | bv1 | Brazil | PA | 2008 | S | S | S | S | S | I | S | 28 | 33 | 2B24 | BE |
| 195 | *B. abortus* | bv3 | Brazil | PA | 2008 | S | S | S | S | S | S | S | 40 | 32 | 2B25 | BG |
| 196 | *B. abortus* | bv3 | Brazil | TO | 2008 | S | S | S | S | S | I | S | 40 | 32 | 2B26 | BH |
| 198 | *B. abortus* | bv3 | Brazil | PA | 2008 | S | S | S | S | S | R | S | 40 | 32 | 2B27 | AC |
| 199 | *B. abortus* | bv3 | Brazil | TO | 2008 | S | S | S | S | S | S | S | 40 | 32 | 2B27 | AC |
| 200 | *B. abortus* | bv3 | Brazil | PA | 2008 | S | S | S | S | S | S | S | 40 | 32 | 2B28 | BI |
| 201 | *B. abortus* | bv3 | Brazil | TO | 2008 | S | S | S | S | S | S | S | 40 | 32 | 2B29 | BJ |
| 202 | *B. abortus* | bv1 | Brazil | TO | 2008 | S | S | S | S | S | S | S | 28 | 34 | 2B30 | BK |
| 203 | *B. abortus* | bv6 | Brazil | PA | 2008 | S | S | S | S | S | S | S | 40 | 2A6 | 2B31 | BL |
| 205 | *B. abortus* | bv1 | Brazil | PA | 2008 | S | S | S | S | S | I | S | 28 | 33 | 2B32 | BN |
| 206 | *B. abortus* | bv3 | Brazil | PA | 2008 | S | S | S | S | S | S | S | 40 | 32 | 2B19 | V |
| 207 | *B. abortus* | bv3 | Brazil | PA | 2008 | S | S | S | S | S | S | S | 40 | 32 | 2B22 | U |
| 208 | *B. abortus* | bv3 | Brazil | TO | 2008 | S | S | S | S | S | S | S | 40 | 32 | 2B22 | U |
| 209 | *B. abortus* | bv2 | Brazil | PA | 2008 | S | S | S | S | S | S | S | 33 | 33 | 52 | BN |
| 211 | *B. abortus* | bv3 | Brazil | TO | 2008 | S | S | S | S | S | S | S | 40 | 32 | 2B33 | T |
| 216 | *B. abortus* | bv3 | Brazil | PA | 2008 | S | S | S | S | S | S | S | 40 | 32 | 2B33 | T |
| 217 | *B. abortus* | bv3 | Brazil | PA | 2008 | S | S | S | S | S | S | S | III | 32 | 2B19 | V |
| 218 | *B. abortus* | bv6 | Brazil | PA | 2008 | S | S | S | S | S | S | S | 40 | 30 | 2B19 | X |
| 219 | *B. abortus* | bv3 | Brazil | PA | 2008 | S | S | S | S | S | S | S | 40 | 32 | 2B16 | BO |
| 223 | *B. abortus* | bv1 | Brazil | MG | 2008 | S | S | S | S | S | S | S | 28 | 33 | 91 | J |
| 224 | *B. abortus* | bv1 | Brazil | MG | 2008 | S | S | S | S | S | S | S | 28 | 33 | 91 | J |
| 227 | *B. abortus* | bv1 | Brazil | MG | NK | S | S | S | S | S | S | S | 28 | 2A7 | 2B52 | DB |
| 233 | *B. abortus* | bv1 | Brazil | MG | NK | S | S | S | S | S | I | S | 28 | 2A9 | 2B53 | DC |
| 234 | *B. abortus* | bv1 | Brazil | MG | NK | S | S | S | S | S | I | S | 28 | 2A7 | 2B53 | DD |
| 235 | *B. abortus* | bv1 | Brazil | MG | NK | S | S | S | S | S | R | S | 28 | 2A7 | 2B48 | DE |
| 236 | *B. abortus* | bv1 | Brazil | MG | NK | S | S | S | S | S | I | S | 28 | 2A7 | 2B48 | DE |
| 237 | *B. abortus* | bv1 | Brazil | MG | NK | S | S | S | S | S | I | S | 28 | 34 | 2B48 | DG |
| 238 | *B. abortus* | bv1 | Brazil | MG | NK | S | S | S | S | S | S | S | 28 | 2A10 | 2B54 | DH |
| 01/06 | *B. abortus* | bv1 | Brazil | RS | 2006 | S | S | S | S | S | I | S | 28 | 33 | 2B4 | D |
| 02/06 | *B. abortus* | bv1 | Brazil | RS | 2006 | S | S | S | S | S | I | S | 28 | 33 | 2B34 | BP |
| 03/06 | *B. abortus* | bv1 | Brazil | RS | 2006 | S | S | S | S | S | I | S | 28 | 33 | 18 | S |
| 07/06 | *B. abortus* | bv1 | Brazil | RS | 2006 | S | S | S | S | S | I | S | 28 | 33 | 18 | S |
| 10/06 | *B. abortus* | bv1 | Brazil | RS | 2006 | S | S | S | S | S | I | S | IV | 34 | 2B13 | BQ |
| 11/06 | *B. abortus* | bv1 | Brazil | RS | 2006 | S | S | S | S | S | I | S | 28 | 33 | 2B35 | BR |
| 13/03 | *B. abortus* | bv1 | Brazil | RS | 2003 | S | S | S | S | S | S | S | 28 | 33 | 2B36 | BS |
| 13a/02 | *B. abortus* | bv1 | Brazil | RS | 2002 | S | S | S | S | S | I | S | 28 | 2A7 | 53 | BT |
| 13b/02 | *B. abortus* | bv1 | Brazil | RS | 2002 | S | S | S | S | S | I | S | 28 | 33 | 53 | BU |
| 14/03 | *B. abortus* | bv1 | Brazil | RS | 2003 | S | S | S | S | S | I | S | 28 | 33 | 18 | S |
| 15/03 | *B. abortus* | bv1 | Brazil | RS | 2003 | S | S | S | S | S | I | S | 28 | 34 | 2B36 | O |
| 16/02 | *B. abortus* | bv4 | Brazil | RS | 2002 | S | S | S | S | S | S | S | 32 | 2A2 | 2B37 | BV |
| 17a/02 | *B. abortus* | bv6 | Brazil | RS | 2002 | S | S | S | S | S | S | S | 40 | 8 | 2B38 | BY |
| 17b/02 | *B. abortus* | bv6 | Brazil | RS | 2002 | S | S | S | S | S | S | S | 40 | 32 | 2B38 | BW |
| 80/04 | *B. abortus* | bv1 | Brazil | RS | 2004 | S | S | S | S | S | I | S | 28 | 33 | 30 | N |
| 89/04 | *B. abortus* | bv1 | Brazil | RS | 2004 | S | S | S | S | S | I | S | 28 | 34 | 30 | M |
| 477 | *B. abortus* | bv1 | Brazil | RS | 1977 | S | S | S | S | S | I | S | 28 | 2A8 | 52 | BZ |
| 577 | *B. abortus* | bv1 | Brazil | RS | 1977 | S | S | S | S | S | S | S | 28 | 2A8 | 53 | CA |
| Ba 56 | *B. abortus* | bv1 | Brazil | RS | 1996 | S | S | S | S | S | I | S | 28 | 33 | 2B39 | CB |
| Ba 96 | *B. abortus* | bv1 | Brazil | RS | 1977 | S | S | S | S | S | I | S | 28 | 33 | 2B40 | CC |
| RS 1 | *B. abortus* | bv1 | Brazil | RS | 2004 | S | S | S | S | S | S | S | 28 | 33 | 2B41 | CD |
| RS2 | *B. abortus* | bv1 | Brazil | RS | NK | S | S | S | S | S | I | S | 28 | 2A7 | 18 | DF |
| RS 3 | *B. abortus* | bv1 | Brazil | RS | 2004 | S | S | S | S | S | I | S | 28 | 33 | 88 | CE |
| RS 4 | *B. abortus* | bv1 | Brazil | RS | NK | S | S | S | S | S | S | S | 28 | 33 | 30 | N |
| RS 5 | *B. abortus* | bv1 | Brazil | RS | 2004 | S | S | S | S | S | S | S | 28 | 2A8 | 18 | CF |
| RS 6 | *B. abortus* | bv1 | Brazil | RS | 2006 | S | S | S | S | S | I | S | 28 | 34 | 64 | CG |
| RS 7 | *B. abortus* | bv1 | Brazil | RS | 2007 | S | S | S | R | S | S | S | 28 | 34 | 53 | CH |
| RS 8 | *B. abortus* | bv1 | Brazil | RS | 2007 | S | S | S | S | S | S | S | 28 | 33 | 29 | CI |
| RS 9 | *B. abortus* | bv1 | Brazil | RS | 2007 | S | S | S | S | S | S | S | 28 | 34 | 66 | CJ |
| RS 10 | *B. abortus* | bv1 | Brazil | RS | NK | S | S | S | S | S | I | S | 28 | 34 | 2B42 | CK |
| SP 1 | *B. abortus* | bv1 | Brazil | SP | NK | S | S | S | S | S | I | S | 28 | 32 | 2B42 | CL |
| SP 2 | *B. abortus* | bv1 | Brazil | SP | NK | S | S | S | S | S | S | S | 28 | 33 | 2B43 | CM |
| SP 3 | *B. abortus* | bv6 | Brazil | SP | NK | S | S | S | S | S | S | S | 40 | 33 | 2B17 | CN |
| SP 4 | *B. abortus* | bv1 | Brazil | SP | NK | S | S | S | S | S | S | S | 28 | 33 | 90 | AD |
| SP 5 | *B. abortus* | bv1 | Brazil | SP | NK | S | S | S | S | S | I | S | 28 | 33 | 2B44 | R |
| SP 6 | *B. abortus* | bv1 | Brazil | SP | NK | S | S | S | S | S | I | S | 28 | 33 | 45 | CO |
| SP 7 | *B. abortus* | bv1 | Brazil | SP | NK | S | S | S | S | S | I | S | 28 | 33 | 91 | J |
| SP 8 | *B. abortus* | bv1 | Brazil | SP | NK | S | S | S | S | S | I | S | 28 | 33 | 2B44 | R |
| SP 9 | *B. abortus* | bv1 | Brazil | SP | NK | S | S | S | S | S | I | S | V | 33 | 2B45 | CQ |
| SP 10 | *B. abortus* | bv1 | Brazil | SP | NK | S | S | S | S | S | I | S | V | 33 | 90 | AE |
| Botucatu | *B. abortus* | bv1 | Brazil | SP | 2007 | S | S | S | S | S | I | S | 28 | 33 | 91 | J |
| GM | *B. abortus* | bv1 | Brazil | SC | 2007 | S | S | S | S | S | I | S | 28 | 33 | 91 | J |
| Pintadinha | *B. abortus* | bv1 | Brazil | SC | NK | S | S | S | S | S | I | S | 28 | 33 | 91 | J |
| Paraí | *B. abortus* | bv2 | Brazil | MG | NK | S | S | S | S | S | S | S | 28 | 33 | 2B46 | K |
| Bawla | *B. abortus* | bv1 | Brazil | SC | NK | S | S | S | S | S | I | S | 28 | 33 | 91 | J |
| 1.1 | *B. abortus* | bv1 | Brazil | SC | NK | S | S | S | S | S | I | S | 28 | 33 | 2B47 | CR |
| 5.1 | *B. abortus* | bv1 | Brazil | MG | NK | S | S | S | S | S | S | S | 28 | 33 | 91 | J |
| 5.2 | *B. abortus* | bv2 | Brazil | MG | NK | S | S | S | S | S | S | S | 28 | 33 | 91 | J |
| 5.3 | *B. abortus* | bv2 | Brazil | MG | NK | S | S | S | S | S | S | S | 28 | 33 | 2B44 | R |
| 6.1 | *B. abortus* | bv1 | Brazil | MG | NK | S | S | S | S | S | I | S | 28 | 33 | 2B48 | L |
| 388.01 | *B. abortus* | bv1 | Brazil | MG | NK | S | S | S | S | S | I | S | 28 | 33 | 2B4 | D |
| 147.6 | *B. abortus* | bv1 | Brazil | MG | NK | S | S | S | S | S | S | S | 28 | 33 | 2B4 | D |
| 393.11 | *B. abortus* | bv1 | Brazil | MG | NK | S | S | R | S | S | I | R | 28 | 33 | 2B46 | K |
| Flor | *B. abortus* | bv2 | Brazil | MG | NK | S | S | S | S | S | S | S | 28 | 33 | 2B46 | K |
| SC01 | *B. abortus* | bv1 | Brazil | SC | NK | S | S | S | S | S | I | S | 28 | 33 | 2B48 | L |
| SC02 | *B. abortus* | bv2 | Brazil | SC | NK | S | S | S | S | S | S | S | 28 | 34 | 52 | CS |
| SC03 | *B. abortus* | bv1 | Brazil | SC | NK | S | S | S | S | S | S | S | 28 | 34 | 2B49 | CT |
| SC04 | *B. abortus* | bv2 | Brazil | SC | NK | S | S | S | S | S | S | S | 28 | 34 | 2B9 | O |
| 20- 180/2M | *B. abortus* | bv1 | Brazil | SP | 2008 | S | S | S | S | S | I | S | 28 | 34 | 30 | M |
| 31- feto144 | *B. abortus* | bv3 | Brazil | SP | 2008 | S | S | S | S | S | I | S | 40 | 8 | 2B50 | CY |
| 24- 192/2M | *B. abortus* | bv6 | Brazil | SP | 2008 | S | S | S | S | S | I | S | 40 | 8 | 2B17 | I |
| 5- 155/2M | *B. abortus* | bv1 | Brazil | SP | 2008 | S | S | S | S | S | I | S | 33 | 8 | 2B17 | I |

^a^CIP: ciprofloxacin; ^b^DOX: doxycycline; ^c^EST: streptomycin; ^d^GEN, gentamicin; ^e^OFX, ofloxacin; ^f^RIF: rifampicin; ^g^SXT, trimethoprim-sulfamethoxazole; ^h^Panel 1; ^i^Panel 2A; ^j^Panel 2B
